# Vestibular evoked myogenic potentials in vestibular migraine and Menière’s disease: cVEMPs make the difference

**DOI:** 10.1007/s00415-020-09902-4

**Published:** 2020-06-03

**Authors:** Julia Dlugaiczyk, Maximilian Habs, Marianne Dieterich

**Affiliations:** 1 German Center for Vertigo and Balance Disorders, University Hospital, Ludwig-Maximilians-Universität, Marchioninistrasse 15, 81377 Munich, Germany; 2 Department of Neurology, University Hospital, Ludwig-Maximilians-Universität, Munich, Germany; 3 Munich Cluster for Systems Neurology (SyNergy), Munich, Germany

**Keywords:** Vestibular evoked myogenic potentials, Menière’s disease, Saccule, Utricle, Vestibular migraine

## Abstract

**Objective:**

Vestibular evoked myogenic potentials (VEMPs) have been suggested as biomarkers in the differential diagnosis of Menière’s disease (MD) and vestibular migraine (VM). The aim of this study was to compare the degree of asymmetry for ocular (o) and cervical (c) VEMPs in large cohorts of patients with MD and VM and to follow up the responses.

**Study design:**

Retrospective study in an interdisciplinary tertiary center for vertigo and balance disorders.

**Methods:**

cVEMPs to air-conducted sound and oVEMPs to bone-conducted vibration were recorded in 100 patients with VM and unilateral MD, respectively. Outcome parameters were asymmetry ratios (ARs) of oVEMP n10p15 and cVEMP p13n23 amplitudes, and of the respective latencies (mean ± SD).

**Results:**

The AR of cVEMP p13n23 amplitudes was significantly higher for MD (0.43 ± 0.34) than for VM (0.26 ± 0.24; adjusted *p* = 0.0002). MD—but not VM—patients displayed a higher AR for cVEMP than for oVEMP amplitudes (MD 0.43 ± 0.34 versus 0.23 ± 0.22, *p* < 0.0001; VM 0.26 ± 0.14 versus 0.19 ± 0.15, *p* = 0.11). Monitoring of VEMPs in single patients indicated stable or fluctuating amplitude ARs in VM, while ARs in MD appeared to increase or remain stable over time. No differences were observed for latency ARs between MD and VM.

**Conclusions:**

These results are in line with (1) a more common saccular than utricular dysfunction in MD and (2) a more permanent loss of otolith function in MD versus VM. The different patterns of o- and cVEMP responses, in particular their longitudinal assessment, might add to the differential diagnosis between MD and VM.

## Introduction

Vestibular evoked myogenic potentials (VEMPs) are short-latency, mainly otolith-driven vestibular reflexes elicited by air-conducted sound (ACS), bone-conducted vibration (BCV), or galvanic vestibular stimulation and recorded from the inferior oblique eye muscle (ocular or oVEMPs) or the sternocleidomastoid muscle (cervical or cVEMPs). While the oVEMP n10 response predominantly represents contralateral dynamic utricular function, the cVEMP p13n23 amplitude is predominantly an indicator of ipsilateral saccular function [1, 2]. Today, VEMPs are widely used as a diagnostic marker for superior canal dehiscence in clinical practice [3]. Their role in the differential diagnosis between Menière’s disease (MD) and vestibular migraine (VM) warrants, however, further research [4].

The current diagnostic criteria for MD and VM developed by the Classification Committee of the Bárány Society are mainly based on patients’ history and symptoms. Depending on the number of fulfilled inclusion criteria, the diagnosis is categorized as “definite” (d) or “probable” (p). In short, MD is defined by at least two recurrent vertigo attacks lasting 20 min to 12 h, audiometrically documented low- to middle-frequency sensorineural hearing loss related to the vertigo attacks, and fluctuating aural symptoms (e.g., tinnitus, aural fullness) in the affected ear [5]. The pathogenesis of MD has been linked to endolymphatic hydrops (ELH) of the inner ear, which appears to be a necessary, but not sufficient condition for the development of MD [6, 7].

On the other hand, the diagnostic criteria for VM include at least five vertigo attacks between 5 min and 72 h, accompanied by at least one migraine symptom (i.e., visual aura, migraine-type headache, photo-/phonophobia) in at least 50% of the attacks and/or a positive history of migraine [8]. The pathophysiology of vestibular migraine is not entirely clear to date. In summary, a combination of central (e.g., a reciprocal connection between the trigeminal and vestibular nuclei, abnormal neurotransmitter modulation in the brainstem) and peripheral pathophysiology (e.g., neurogenic inflammation of the inner ear mediated by projections of the trigeminovascular system to the labyrinthine artery) most likely explains the various clinical presentations of this multi-facetted disorder [9, 10].

Despite the progress in classification of vestibular disorders, the differential diagnosis between MD and VM in clinical practice is often difficult due to the episodic and fluctuating nature of both diseases and the considerable overlap of symptoms and, eventually, diseases [11, 12]. Therefore, biomarkers to distinguish between the two disorders would be highly valuable. As a first step, it is important to identify possible markers that differ between patients and healthy subjects. A number of studies have compared VEMPs from patients with either MD (e.g., [15, 16]) or VM (e.g., [20, 21]) to healthy controls, indicating their diagnostic utility for both disorders. As a second step, the biomarker should aid the differential diagnosis between MD and VM in patients whose history and symptoms cannot be clearly attributed to one of the disorders [12]. Studies comparing the different patterns of o- and cVEMP responses between patients with MD and VM are, however, scarce.

Concerning absolute amplitudes, Baier and Dieterich [23] reported no difference for ACS cVEMPs in VM versus MD; Zuniga et al. [24] observed reduced amplitudes for ACS oVEMPs in unilateral MD as compared to VM. Studies comparing VEMP asymmetry ratios (ARs) include Inoue et al. [25], who described higher ACS oVEMP ARs in unilateral MD (77%) versus VM (57%), but equal asymmetry ratios for BCV oVEMPs (27% versus 34%) and ACS cVEMPs (51% versus 44%). On the other hand, Taylor et al. [26] and Salviz et al. [27] reported a significantly higher AR for ACS cVEMP amplitudes in patients with unilateral MD (46% and 29%) as compared to those with VM (16% in both studies). Despite some inconsistent results, the AR studies generally indicated that VEMP amplitudes of MD patients were more asymmetric than those in VM. None of these studies, however, included more than 60 patients with each disorder or performed VEMP recordings at different time points. Furthermore, the diagnostic criteria of the Bárány Society for MD and VM were not applied in all of the studies; in particular, none of them distinguished between possible and definite MD.

Therefore, the primary aim of the present study was to test whether c- and oVEMPs displayed different degrees of asymmetry in a large cohort of consecutive, unselected patients with MD or VM according to the Bárány Society classification criteria. Second, we investigated whether these results allow “definite” and “probable” disease categories of MD and VM to be distinguished from each other. Third, we analyzed in some cases whether the episodic character of VM and MD is reflected by fluctuating VEMP responses obtained at different points in time.

## Materials and methods

### Study subjects

For this retrospective study, the medical records of consecutive patients with VM or MD, who had received VEMP testing at the German Center of Vertigo and Balance Disorders or the Department of Neurology at the University Hospital of the Ludwig-Maximilians-Universität, Munich, Germany, in 2012–2013 and 2017–2019, were screened. One hundred (100) patients were included in the MD and the VM group, respectively. The MD group comprised 85 patients with definite MD (dMD) and 15 patients with probable MD (pMD), while the VM group consisted of 35 patients with definite VM (dVM) and 65 patients with probable VM (pVM). In addition to those patients, four MD and five VM patients, who had received serial VEMP measurements over time, were identified and analyzed separately.

All subjects underwent a thorough neurotological workup, i.e., history-taking, clinical examination, and additional vestibular diagnostics, including caloric irrigation of the horizontal canals, video head impulse testing for the semicircular canal function in the high-frequency range, examination of the subjective visual vertical and fundus photography with a scanning laser ophthalmoscope for static graviceptive function. In addition, c- and oVEMPs, posturography, gait analysis, and pure tone audiometry were performed if necessary.

Patients were included in the study if they had received VEMP testing and fulfilled the diagnostic criteria of the Bárány Society for either vestibular migraine [8] or Menière’s disease [5]. All patient records were reviewed by a neurotology specialist for the presence of the diagnostic criteria defining the definite and probable forms of the disorders before inclusion in the study. Patients were excluded from the study if they had reported overlapping symptoms of MD and VM or if they fulfilled the diagnostic criteria for both disorders based on their history.

Moreover, the following groups of patients were excluded: bilateral MD, MD with a history of migraine, patients with further vestibular or neurological disorders (e.g., benign paroxysmal positional vertigo, vestibular paroxysmia, inner and outer labyrinthine fistula, vestibular neuritis, vestibular schwannoma, cerebellar ataxia, extrapyramidal motor disorders, dementia, multiple sclerosis, stroke), middle ear disease (e.g., cholesteatoma, otosclerosis, chronic otitis media, tympanic effusion), or an air–bone gap in pure tone audiometry on the day of the VEMP recording. Furthermore, patients with a history of ear surgery (including but not limited to tympanoplasty, stapes surgery, endolymphatic sac surgery, intratympanic gentamicin, and labyrinthectomy), brain surgery, or concussion were excluded.

### VEMP recordings

VEMPs were recorded as described previously [1] with one of the three VEMP platforms routinely used in the German Center for Vertigo and Balance Disorders and the Department of Neurology, i.e., the Nicolet on Viking EDX evoked potential system (Natus, Pleasanton, CA, USA), the Eclipse platform (Interacoustics, Middelfart, Denmark), or the Neuropack M1 platform (Nihon Kohden, Tokyo, Japan). Only those VEMP responses that were clearly discernible from background noise were included in the analysis. To avoid bias due to different recording platforms and examiners, only asymmetry ratios of VEMP amplitudes and latencies were analyzed in detail (see “Outcome parameters”).

#### oVEMPs

For oVEMPs, the surface recording electrode was placed on the infraorbital rim, the reference electrode 1–2 cm below, and the ground electrode was fixed around the wrist. Patients lay supine and looked up during the recording to increase the oVEMP n10 amplitude [2]. A BCV stimulus was delivered to the midline of the forehead at the hairline (Fz) by a powerful bone-conduction device (minishaker 4810, Bruel and Kjaer, Naerum, Denmark) connected to an amplifier (type 2718, Bruel and Kjaer, Naerum, Denmark). A custom-made Matlab program (MathWorks, Natick, MA, USA) was used to produce 500 Hz tone burst BCV stimuli (rise/fall time: 0 ms, plateau: 2 ms, driving voltage: 5 V, stimulus repetition rate: 3 pulses per second (pps)). Responses below the right and left eyes were recorded simultaneously; the analysis window was 20 ms from stimulus onset. The EMG signal was amplified and bandpass filtered (10 Hz – 1.5 kHz). Twenty unrectified traces were averaged per recording, and at least two trials were run in one subject to ensure reliability and reproducibility of the VEMP signal. Amplitudes and latencies from reproducible recordings were averaged for the right and left ear, respectively (see “Outcome parameters”).

#### cVEMPs

For cVEMPs, the recording electrode was placed over the mid third of the sternocleidomastoid muscle (SCM), and the reference electrode over the sternoclavicular junction. The ground electrode was fixed around the wrist. During the recording, the subject lifted the head in the midline from a semi-recumbent position to maintain sufficient symmetric muscular activity in the SCM of either side for recording the inhibitory cVEMP response [2]. 500 Hz ACS tone burst stimuli (130 dB peak sound pressure level, rise/fall time: 1 ms, plateau: 5 ms, 3 pps) were applied consecutively to either ear by TDH-39P headphones (Telephonics, Framingdale, NY, USA). The recording window was 50 ms from stimulus onset. The EMG signal was amplified and bandpass filtered (10 Hz – 2 kHz), and 50 unrectified traces were averaged per recording. As described for oVEMPs above, amplitudes and latencies were calculated as average from at least two reproducible recordings.

### Outcome parameters

For oVEMPs, amplitudes and latencies of the first negative (n10) and the first positive (p15) peak were determined as a measure of contralateral (mainly) utricular function, while the first positive (p13) and the first negative peak (n23) of the cVEMP response were used as parameters for ipsilateral (mainly) saccular function [2].

To rule out any systematic differences between the three different sets of recording equipment and different examiners, we did not compare absolute amplitudes and latencies between subjects, but only ARs between the right and left sides within each subject. Amplitude ARs were calculated as described before [28]:

$${\text{AR}}\, = \left| {{\text{ right amplitude }}{-}{\text{ left amplitude }}} \right|{\text{ }}{ / }{\text{ }}\left| {{\text{ right amplitude}}\, + \,{\text{left amplitude }}} \right|.$$

AR values range between 0 (symmetric response on both sides) and 1 (absent response on one side). Asymmetry ratios were determined for oVEMP n10p15 amplitudes and cVEMP p13n23 amplitudes. ARs > 0.3 (oVEMPs) and ARs > 0.4 (cVEMPs) were considered to indicate asymmetry between the two sides, respectively, based on the normative values of our laboratory as well as data from the literature [2, 28].

Latency ARs were calculated accordingly for n10 and p15 latencies (oVEMPs) and p13 and n23 latencies (cVEMPs) in addition to n10p15 and p13n23 inter-peak intervals. All values are presented in mean ± standard deviation (SD).

### Data analysis

Data were entered into an Excel 2013 spreadsheet (Microsoft, Redmond, WA, USA) and analyzed with GraphPad Prism 8.3.1 software (GraphPad, San Diego, CA, USA). For all statistical tests, an (adjusted) *p* value < 0.05 was considered to indicate a statistically significant difference. Age between the MD and VM groups was compared using the two-sided unpaired Student’s *t* test, gender distribution between the two groups was analyzed with the two-sided Fisher’s exact test. Simple linear regression was employed to determine a possible correlation between VEMP ARs and age, and a *p* value < 0.05 was considered to indicate a slope significantly different from zero. For the comparison of amplitude ARs between different groups, we used the Brown-Forsythe and Welch ANOVA test, as SDs were significantly different for the individual groups. ANOVA was followed by Dunnett’s T3 test to correct for multiple comparisons. Finally, latency ARs between the MD and the VM groups were analyzed by two-sided unpaired *t* tests corrected for multiple comparisons.

## Results

### Demographics

Individuals with MD were significantly older (58.85 ± 13.85 years) than those with VM (36.03 ± 19.49 years) (two-sided unpaired *t* test: *p* < 0.0001; Table 1). The female proportion was significantly lower in MD (38%) as compared to VM (70%) (two-sided Fisher’s exact test: *p* < 0.0001).

**Table 1:** Subjects’ demographic information

	MD (*n* = 100)	VM (*n* = 100)	*p* value
Age (years; mean ± SD)	58.85 ± 13.85	36.03 ± 19.49	*p* < 0.0001 (two-sided unpaired *t* test)
Female: male ratio	38: 62 (0.6: 1)	70: 30 (2.3: 1)	*p* < 0.0001 (two-sided Fisher’s exact test)

*MD*  Menière’s disease, *VM*  vestibular migraine

### Asymmetry ratio of VEMP amplitudes

**Fig. 1:**
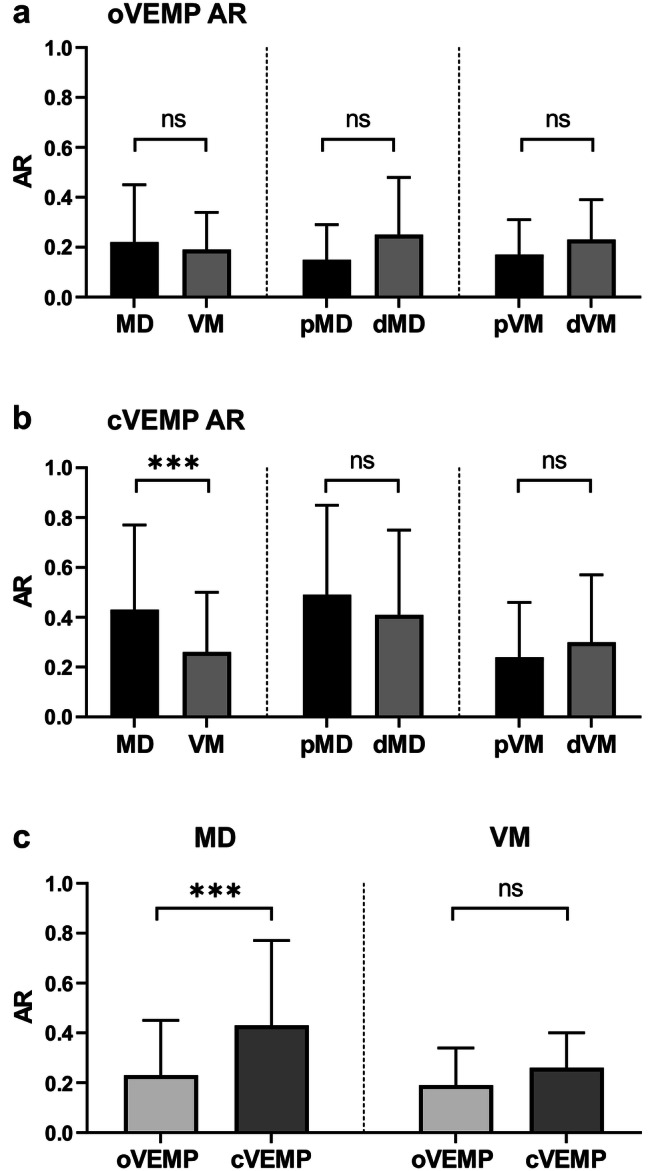
Asymmetry ratio (AR) for ocular and cervical vestibular evoked myogenic potentials (o- and cVEMPs) in Menière’s disease (MD) and vestibular migraine (VM) (mean + standard deviation (SD), *ns* not significant, ***adjusted *p* value < 0.001). **a** Comparison of oVEMP ARs between MD and VM, probable versus definite MD (pMD/dMD) and probable versus definite VM (pVM/dVM). No difference was observed between the groups. **b** Comparison of cVEMP ARs between MD and VM, probable versus definite MD (pMD/dMD) and probable versus definite  VM (pVM/dVM). cVEMP amplitude AR was significantly higher in MD than in VM (adjusted *p* value = 0.0002). **c** Comparison of o- versus cVEMP amplitude ARs in MD and VM. cVEMP amplitude AR was higher than oVEMP amplitude AR for MD (adjusted *p* value < 0.0001), but not for VM patients

Asymmetry ratios of oVEMP n10p15 and cVEMP p13n23 peak-to-peak amplitudes (oVEMP and cVEMP amplitude ARs) are shown in Fig. 1.

First, ARs between MD and VM were compared. No significant statistical difference was observed for the oVEMP amplitude AR (Fig. 1a) between the MD (0.23 ± 0.22) and the VM group (0.19 ± 0.15) (ANOVA with multiple comparisons: adjusted *p* value = 0.35). In addition, the oVEMP amplitude AR did not discriminate between those subjects with probable versus definite MD (pMD 0.15 ± 0.14, dMD 0.25 ± 0.23; adjusted *p* = 0.09) and probable versus definite VM (pVM 0.17 ± 0.14, dVM 0.23 ± 0.16; adjusted *p* = 0.93). Neither the MD nor the VM group showed a correlation between age and oVEMP amplitude ARs (simple linear regression; MD: *p* = 0.93; VM: *p* = 0.67).

On the other hand, cVEMP amplitude AR (Fig. 1b) was significantly higher for subjects with MD (0.43 ± 0.34) as compared to those with VM (0.26 ± 0.24) (ANOVA with multiple comparisons: adjusted *p* value = 0.0002). As observed for oVEMPs, cVEMP amplitude ARs did not differ between probable and definite MD (pMD 0.49 ± 0.36, dMD 0.41 ± 0.34; adjusted *p* = 0.81) and between probable and definite VM subgroups (pVM 0.24 ± 0.22; dVM 0.30 ± 0.27; adjusted *p* = 0.60). No correlation between cVEMP amplitude AR and age was observed for both disorders (simple linear regression; MD *p* = 0.51; VM *p* = 0.39).

Second, we determined the degree of asymmetry for o- versus cVEMPs within one group of patients (MD or VM, Fig. 1c). For MD, the amplitude AR was significantly higher for c- than for oVEMPs (0.43 ± 0.34 versus 0.23 ± 0.22; adjusted *p* value < 0.0001), while there was no difference between c- and oVEMP ARs for patients with VM (cVEMPs 0.26 ± 0.14; oVEMPs 0.19 ± 0.15; adjusted *p* value = 0.11).

Third, cVEMP ARs in MD were further analyzed with respect to the affected ear. Only three patients displayed an increased p13n23 amplitude on the affected side. All of them had experienced audio-vestibular symptoms typical of MD for less than 2 years. On the other hand, those MD patients with reduced cVEMP amplitudes on the affected side had a mean disease duration of 8.6 years.

In summary, (1) the mean cVEMP amplitude AR was higher for MD than for VM patients, (2) patients with MD had a significantly higher mean amplitude AR for cVEMPs than for oVEMPs, and (3) increased cVEMP amplitudes on the affected side were only observed in early stages of MD.

### Asymmetry ratio of VEMP latencies

Asymmetry ratios were calculated for peak latencies (oVEMP n10 and p15; cVEMP p13 and n23) and inter-peak intervals (oVEMP n10p15; cVEMP p13n23) and are summarized in Table 2. No statistically significant difference was observed for all peak latencies and inter-peak intervals between the MD and the VM groups (two-sided unpaired *t* test corrected for multiple comparisons; all adjusted *p* values > 0.05).

**Table 2:** Asymmetry ratios (ARs) of peak and inter-peak latencies for ocular and cervical vestibular evoked myogenic potentials in subjects with Menière’s disease (MD) and vestibular migraine (VM), mean ± SD

	MD (*n* = 100)	VM (*n* = 100)	Adjusted *p* value
AR n10 peak latency	0.03 ± 0.03	0.03 ± 0.04	> 0.99
AR p15 peak latency	0.03 ± 0.02	0.03 ± 0.02	> 0.99
AR n10p15 interval	0.09 ± 0.08	0.08 ± 0.07	0.72
AR p13 latency	0.05 ± 0.05	0.04 ± 0.04	0.47
AR n23 latency	0.04 ± 0.04	0.03 ± 0.03	0.25
AR p13n23 interval	0.11 ± 0.14	0.09 ± 0.07	0.60

An adjusted *p* value < 0.05 in the two-sided unpaired *t* test corrected for multiple comparisons was considered to indicate a significant difference between groups

### Monitoring of VEMPs in single patients

**Fig. 2:**
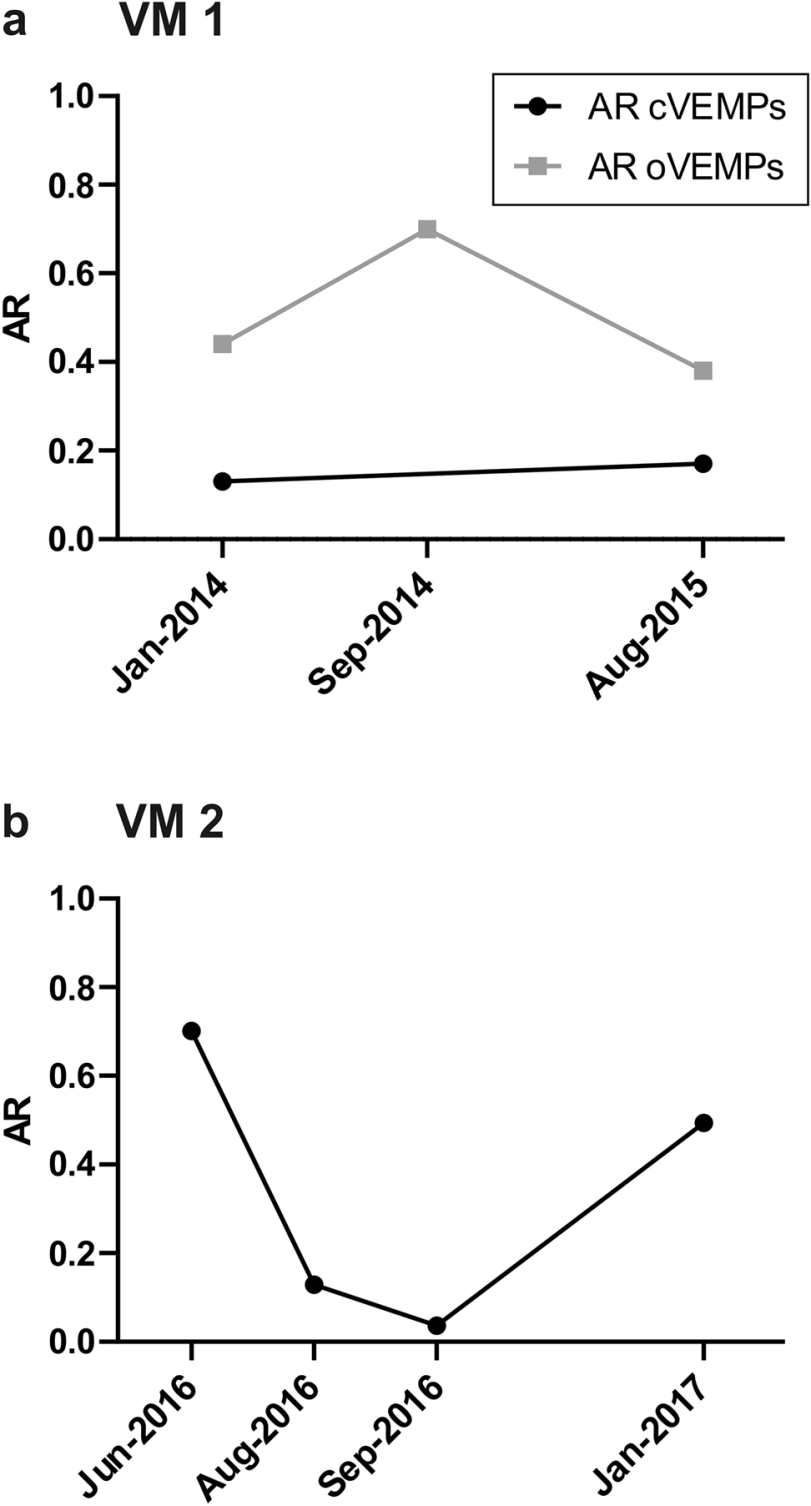
Serial measurements of the asymmetry ratio for ocular vestibular evoked myogenic potentials (AR oVEMPs, grey squares) and cervical vestibular evoked myogenic potentials (AR cVEMPs, black circles) in two patients with vestibular migraine (VM 1 and VM 2) showing either fluctuating or stable degrees of asymmetry. See Table 3 for a summary of the patients’ clinical data

**Fig. 3:**
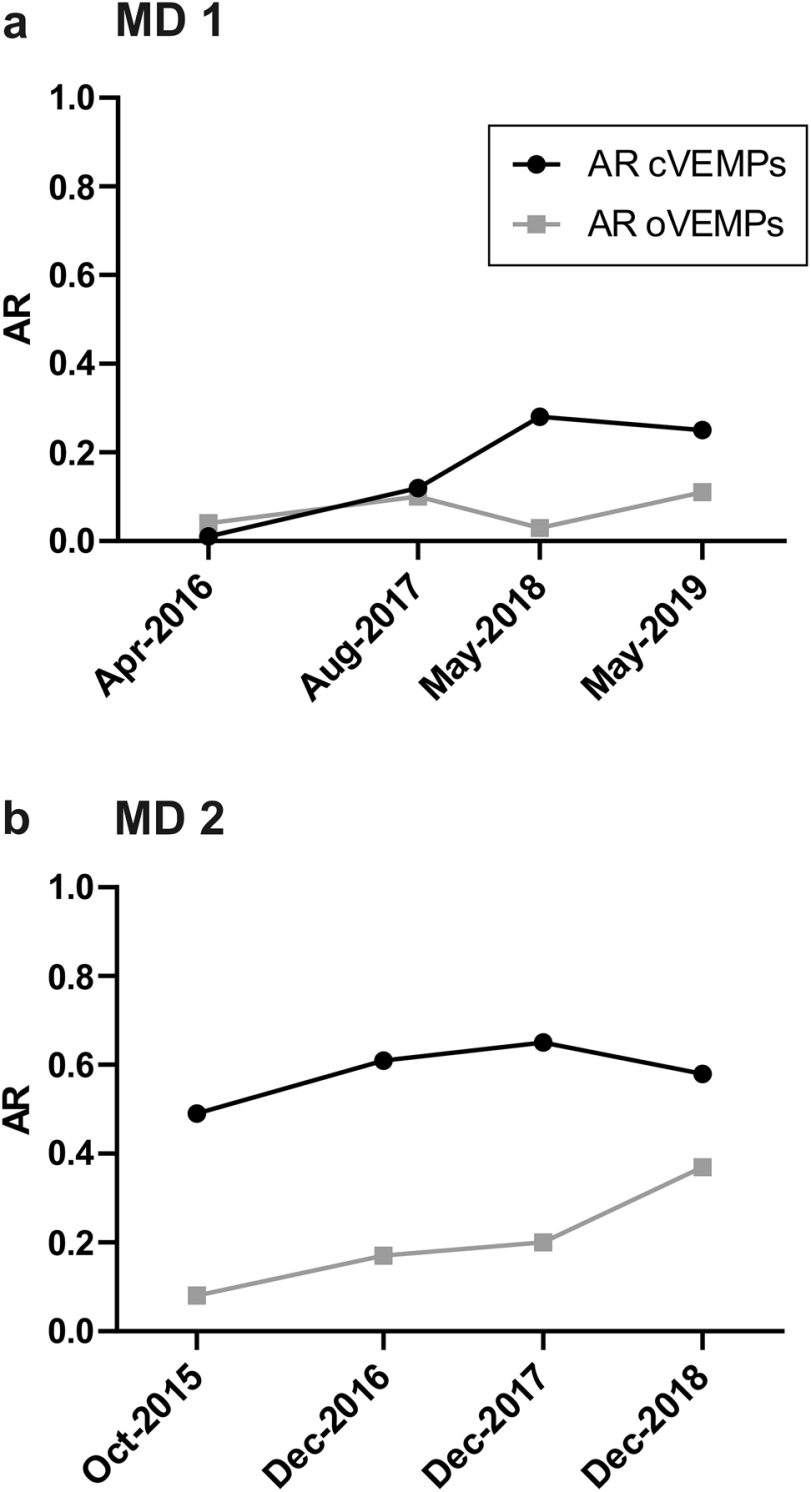
Serial measurements of the asymmetry ratio for ocular vestibular evoked myogenic potentials (AR oVEMPs, grey squares) and cervical vestibular evoked myogenic potentials (AR cVEMPs, black circles) in two patients with Menière’s disease (MD 1 and MD 2) showing either increasing or stable ARs. See Table 3 for a summary of the patients’ clinical data

For five VM and four MD patients, multiple VEMP recordings from follow-up visits were available. Due to the small number of patients and the inter-individual differences in disease duration/activity and follow-up intervals, no statistical analysis was performed. Figures 2 and 3 show the development of o- and cVEMP amplitude ARs over time in those patients in whom data from at least three different points in time were available. The basic clinical information (e.g., symptoms, number of attacks, results of vestibular testing) of these patients is summarized in Table 3.

**Table 3:** Clinical data in patients with follow-up recordings of vestibular evoked myogenic potentials (VEMPs) from Figs. 2 and 3

	Diagnosis and gender	Disease duration (years)	Symptoms		Audio-vestibular test results	
		Baseline	Baseline	Follow-up	Baseline	Follow-up
VM 1	pVM f	7	Spontaneous and positional vertigo attacks for minutes, clustering over several days; 1x/month; accompanying symptoms: nausea, vomiting, photo-/phonophobia, visual aura, sometimes frontal headache; no history of migraine	Treatment with propranolol (5 mg TID); same frequency of attacks, but reduced severity	SVV tilt to the left (5°), normal PTA, normal calorics, normal vHIT	Recovery of SVV tilt, slightly saccadic smooth pursuit
VM 2	dVM m	1	Spontaneous vertigo attacks (ca. 15 min), 2-3x/month; accompanying symptoms: nausea, frontal headache, photophobia; positive migraine history	Moderate daily physical activity (30 min); 0–1 attacks/month from Aug to Oct 2016; 2 attacks/month from Nov 2016 to Jan 2017	Normal PTA, normal vHIT, right HSN, no hydrops in inner ear MRI	Variable caloric asymmetry (first right, then left hypo-excitability), no HSN
MD 1	dMD left m	1	Spontaneous vertigo attacks (up to 12 h), 2x/week; accompanying symptoms: nausea, ear fullness, tinnitus, hearing loss on the left	Apr 2016: patient treated with betahistine dihydrochloride 144 mg TID since Dec 2015, no attacks. Temporary increase in attack frequency in May 2016 (2x/week) and Jan 2017 (1–2×/month), progressive hearing loss and chronic tinnitus on the left. ⇒ Symptom-based adjustment of medication (up to 288 mg betahistine TID) Aug 2017–May 2019: no vertigo attacks, sometimes imbalance. ⇒ Betahistine 96 mg TID	PTA: low-frequency hearing loss on the left, calorics: hypo-excitability on the left (42%), normal vHIT	PTA: progressive low-frequency hearing loss on the left, calorics: progressive hypoexcitability on the left (60%)
MD 2	dMD left m	8	Spontaneous vertigo attacks (hours) with ear fullness, tinnitus and hearing loss on the left; Tumarkin drop attacks (seconds) without accompanying ear symptoms	Oct 2015–Dec 2017: patient treated with betahistine dihydrochloride 24 mg TID, no vertigo attacks, no Tumarkin attacks, occasional imbalance. ⇒ Betahistine 24 mg BID Dec 2017–Oct 2019: 3 short attacks with vertigo or leftward sway (sec–min), no falls, progressive hearing loss and tinnitus on the left. ⇒ Betahistine 96 mg TID	PTA: low-frequency hearing loss on the left, normal calorics, normal vHIT	PTA: pantonal hearing loss on the left, normal calorics, normal vHIT

*BID* two times a day, *dMD* definite Menière’s disease, *dVM* definite vestibular migraine, *f* female, *HSN* head-shake nystagmus, *m* male, *MRI* magnetic resonance imaging, *pVM* probable vestibular migraine, *PTA* pure tone audiogram, *SVV* subjective visual vertical, *TID* three times a day, *vHIT* video head impulse test

Patient VM 1 (Fig. 2a) had a 7-year history of pVM at the beginning of the monitoring (January 2014) and displayed a fluctuating level of oVEMP ARs (0.38–0.70), while the cVEMP AR was relatively low and stable (< 0.2) over the next 18 months. Patient VM 2 (Fig. 2b), on the other hand, had suffered from dVM for only 1 year at the time of the first cVEMP measurement in June 2016. Here, highly variable cVEMP amplitude ARs ranging between 0.13 and 0.70 were observed between June 2016 and January 2017.

Two patients with dMD (MD 1 and MD 2) were followed over 3 years each. For both patients, o- and cVEMP amplitudes recorded from the affected labyrinth were smaller than or equal to those from the contralateral side. Patient MD 1, who presented with a 1-year history of left-sided MD in April 2016, showed a slow increase in cVEMP amplitude AR (0.01–0.28) followed by a plateau, while the oVEMP AR remained < 0.1 for 3 years (Fig. 3a). Patient MD 2 had experienced MD vertigo attacks with accompanying auditory symptoms and Tumarkin drop attacks for 8 years when he had his first examination in October 2015. His cVEMP amplitude AR was stable around 0.6 with a slight increase over time, while the oVEMP amplitude AR increased between 2015 and 2018 from 0.08 to 0.37 (Fig. 3b).

## Discussion

The present study compared c- and oVEMP responses in large cohorts of 100 patients with MD and VM, respectively, from an interdisciplinary tertiary center for vertigo and balance disorders. The most important findings were (1) the asymmetric cVEMP response pattern for MD versus VM, (2) the higher degree of cVEMP as compared to oVEMP amplitude asymmetry for MD, and (3) variable degrees in asymmetry for VEMP responses in both MD and VM patients over time.

### VEMP responses in Menière’s disease

The higher cVEMP amplitude AR for unilateral MD patients versus VM patients in the present study (Fig. 1b) confirms the findings by Taylor et al. [26] and Salviz et al. [27] in smaller patient groups and emphasizes a pivotal role of the saccule in the pathogenesis of MD. The high AR may either result from increased ACS cVEMP amplitudes on the affected side in early stages of the disease, which have been attributed to the distended saccular membrane contacting the stapes footplate, or from reduced amplitudes on the affected side in later stages of the disease [16, 29, 30], probably due to a progressive loss of vestibular hair cells and primary vestibular neurons [33, 34]. In line with this, the three MD patients with an increased cVEMP amplitude on the affected side in our study had experienced audio-vestibular symptoms for less than 2 years, whereas those with a reduced amplitude had a mean disease duration of 8.6 years. Histopathological analyses in MD have shown that the saccule is affected more often by ELH than the utricle [35]. This finding is reflected by the higher degree of asymmetry for cVEMP as compared to oVEMP amplitudes in the MD patients of our study (Fig. 1c).

The discrepancy in the ARs of c- and oVEMPs for MD observed in the present study is also noteworthy with respect to inner ear neurophysiology, as it implies that ACS cVEMPs and BCV oVEMPs originate in two different subsets of vestibular hair cells, namely those of the saccule and the utricle [36]. Furthermore, the different asymmetry ratios for o- and cVEMPs in MD indicate that the endolymphatic volumes in the utricle and in the saccule are regulated independently from each other. The utriculo-endolymphatic valve (Bast’s valve) at the junction between the utricle and utricular duct seems to play an important role in this process [37]. Studies in guinea pigs have shown that saccular hydrops compresses the utricular duct and closes Bast’s valve, thus allowing normal endolymphatic pressure to be maintained in the utricle independent of endolymphatic volume and pressure in the saccule [16, 38].

The low degree of asymmetry for BCV oVEMP amplitudes of MD patients in the present study (Fig. 1a) confirms the results from Inoue et al. [25]. On the other hand, a higher AR for ACS oVEMP amplitudes was observed for MD than VM in the latter study. One probable reason for this discrepancy might be the fact that BCV is generally a more powerful stimulus than ACS. Therefore, mild utricular damage might not be detected by BCV, whereas it might be sufficient to cancel the small ACS-induced response [24, 25].

Amplitude ARs in the present study did not allow discrimination between probable and definite forms of MD (Fig. 1a, b). For oVEMP amplitude ARs, there was a tendency towards a higher degree of asymmetry in dMD than pMD, which would be in line with a more common involvement of the utricle in advanced stages of the disease. The results did, however, not reach the level of statistical significance in multiple comparisons, which might be due to the low number of patients in the pMD group (*n* = 15). Therefore, a larger number of patients with pMD should be examined in future studies to further analyze a possible progression of utricular damage from probable to definite MD.

### VEMP responses in vestibular migraine

In general, the low and similar degrees of asymmetry for o- and cVEMP amplitudes in VM (Fig. 1c) indicate a common central pathology affecting the utricle and the saccule of both labyrinths equally. Possible mechanisms include the reciprocal connection between the trigeminal and vestibular nuclei and an abnormal neurotransmitter modulation in the vestibular nuclei. Furthermore, the trigeminovascular system projects to the labyrinthine arteries of either labyrinth, which might result in symmetrical involvement of both labyrinth organs in VM [9]. The symmetrical responses for o- and cVEMPs in VM fit with the recent observation of mild bilateral ELH in inner ear magnetic resonance imaging (MRI) of patients with VM [39].

In this context, it is important to note that the degree of the mild bilateral ELH fluctuated over time in the latter case report. In line with this finding, we detected fluctuating degrees of asymmetry for o- and cVEMP amplitudes in serial examinations of VM patients in the present study (Fig. 2). In particular, the high degrees of ARs up to 0.7 point to a temporary asymmetry between right- and left-sided otolith function, which might be due to temporary unilateral hypoperfusion of the labyrinthine artery [10] or an asymmetric ELH. In summary, the VEMP response pattern of VM patients in the present study strengthens the notion that this disorder is caused by a combination of fluctuating (and not persisting) peripheral and central pathologies [20].

As observed for MD above, VEMP amplitude ARs were not able to discriminate between probable and definite cases of the disease (Fig. 1a, b). This is in line with the notion that VM is an episodic disorder that does not result in permanent damage of the vestibular organs in the inner ear.

Regarding the ARs for o- and cVEMP latencies, there was no difference between MD and VM in the present study (Table 2). Although this finding does not allow any conclusions about absolute latencies, it indicates that the central pathology in VM does most likely not involve unilateral demyelination in the brainstem, which has been linked to prolonged VEMP latencies [21].

### Diagnostic utility of VEMPs in distinguishing Menière’s disease from vestibular migraine

The paramount aim of the present study was to determine whether different patterns of o- and cVEMP responses in MD and VM might be helpful for the differential diagnosis between the two disorders. Of all the parameters analyzed, only the cVEMP amplitude AR yielded a statistically significant difference between the two groups (Fig. 1b: mean = 0.43 ± 0.34 for MD and 0.26 ± 0.24 for VM). This parameter is, however, only of limited value in clinical practice due to the broad range and the overlap of results for the two disorders in this study.

It has been recommended to combine the inter-aural cVEMP amplitude AR for 500 Hz ACS with the 500–1000-Hz frequency tuning ratio of either ear to increase the diagnostic accuracy of VEMPs in differentiating MD patients from healthy subjects and VM patients [19, 26]. Monitoring of VEMP responses over time might be another promising approach to achieve this aim.

### Monitoring of VEMPs in single patients

In this retrospective study, VEMP responses from at least three different points in time were only available for two VM and two MD patients (Figs. 2, 3), as VEMP monitoring was not routinely performed in our vertigo clinic in each patient. Although the informative value of these data is very limited at the moment, a systematic analysis of VEMP responses over time in MD and VM patients, particularly those with overlapping symptoms, might be useful in future clinical studies.

The most important finding in this study was the variability in VEMP amplitude ARs over time for both disorders. It should be particularly noted that o- and cVEMP ARs change independently within one patient (e.g. Fig. 3a) adding further evidence to the notion that o- and cVEMPs originate in two different subsets of vestibular receptors, i.e., the utricle and the saccule [36].

The longitudinal development of VEMP responses was studied in MD patients in more detail during the last 20 years. Fluctuating o- and cVEMP amplitudes were recorded in the early stages of MD disease, particularly shortly before and after an attack [29, 36], while patients with long-standing disease typically displayed decreasing VEMP amplitudes on the affected side and thus increasing ARs, reflecting a progressive permanent damage to the sensory epithelia of the labyrinth [16, 32]. In line with this, patient MD 1 with a 1-year history displayed an increase in cVEMP amplitude ARs within the next 3 years due to decreasing amplitudes on the affected side (Fig. 3a). The lower AR values for o- versus cVEMPs in this patient reflect the mean results for the whole MD group and fit with a predominance of saccular rather than utricular dysfunction. Patient MD 2, on the other hand, had experienced both MD vertigo and Tumarkin attacks for 8 years (Fig. 3b). Tumarkin attacks, which occur in about 5% of MD patients, are considered to arise from abrupt changes of endolymphatic pressure in the otolith organs. Based on histopathological and VEMP studies, Tumarkin attacks are thought to be triggered by residual utricular function, while structural damage and absent cVEMPs indicated severe saccular dysfunction [16]. In accordance with results from a previous study [40], the relatively stable cVEMP amplitude AR of 0.6 (Fig. 3b) was due to reduced amplitudes on the affected (left) side, indicating chronic saccular dysfunction on the left, while the slightly increasing oVEMP amplitude AR fits with residual, but deteriorating left-sided utricular dysfunction. Furthermore, patient MD 2 had a normal caloric response (horizontal semicircular canal function) on the affected side (Table 3), as observed before in 40% of patients with Tumarkin attacks [40].

The issue of temporal changes in VEMP responses has, however, received less attention in VM so far. To the best of our knowledge, only one study has compared cVEMP recordings between patients with MD and VM at two different points in time. Van Tilburg and coworkers [41] observed an increase in cVEMP amplitude AR after a mean follow-up period of 2 years in MD patients, while no significant changes were observed in VM patients. Although the results of the present study are very preliminary due to the small sample size, they indicate variable degrees of asymmetry over time (in particular in patient VM 2 with a short disease history), in contrast to the steady increase in AR observed for the two MD patients (Figs. 2, 3). This aspect warrants further research. Fluctuating peripheral and central vestibular dysfunctions in VM were also reflected by the variable degrees of inter-ictal ocular motor abnormalities in patient VM 1 and the caloric asymmetry in patient VM 2 (Table 3). Such findings are quite commonly observed in VM [11].

### Strengths and limitations of the present study

#### Patient demographics

Overall, patients with VM were younger than those with MD in the present study, reflecting the earlier mean age of disease onset for patients with VM as compared to MD [12]. It is very unlikely that the higher AR for cVEMP amplitudes in MD is due to the older age of these patients, as ARs do not correlate with age in the present study, for either the MD or the VM group.

Moreover, the female proportion was higher in VM as compared to MD in our patients, which is in line with a recent meta-analysis on this topic [42]. Thus, the present study comprises representative samples of MD and VM in terms of age and gender.

#### Strengths

To the best of our knowledge, this is the first study comparing o- and cVEMPs between patients with MD and VM using the Bárány Society classifications for both disorders, whereas previous studies applied the Neuhauser [43] or Bárány Society criteria [8] for VM and the criteria of the American Academy for Otolaryngology-Head and Neck Surgery (AAO-HNS) for MD [44]. In contrast to other studies, which only differentiated between probable and definite VM (e.g., [23, 26]), we included a separate analysis of VEMPs in patients with pMD and dMD. Thus, this study helps to implement the Bárány Society’s International Classification of Vestibular Disorders in clinical research, which will hopefully facilitate the comparison between different studies in the future.

Moreover, this is the largest study published so far comparing VEMPs in patients with MD and VM (*n* = 100 patients each). Finally, we provide a first insight into the longitudinal development of VEMP amplitude ARs in VM, an aspect that has been neglected so far.

#### Limitations

There are, however, several limitations to the present study. First, this is a retrospective study from a tertiary neurotology center, which might result in a selection bias towards patients with more severe forms of MD and VM. In addition, disease duration/activity, treatment and follow-up intervals differed between patients and groups. A further limitation is the fact that we had to rely on patients’ history from the medical records to differentiate between MD and VM, and between probable and definite forms of the disorders. This information may be incomplete and subject to recall bias.

Second, our aim of reflecting everyday clinical practice in a large vertigo clinic means that patients were examined by different examiners and with different measurement setups. To minimize the risk of systematic differences between examiners and sets of equipment, we did not compare absolute VEMP amplitudes and latencies between patients, but only asymmetry ratios comparing amplitudes and latencies within one subject. Furthermore, only those serial measurements from one patient that had been recorded with the same setup were included in the study, which limited the number of follow-up measurements available for analysis.

### Outlook

The observation of fluctuating VEMP responses in VM should be analyzed systematically over several years in further studies. In particular, the correlation of VEMP amplitudes/AR with clinical symptoms and inner ear ELH deserves special attention. For MD, both the degree of ELH in the affected ear [45, 46] and the AR of cVEMP amplitudes increase over time [32, 41], which is in line with progressive permanent damage to the sensory epithelia of the labyrinth. Assuming that VM does not result in permanent hair cell damage or neuronal degeneration of the inner ear, we hypothesize that VEMP amplitudes/AR and the degree of ELH would fluctuate over time without progressive deterioration as observed for MD. Thus, the different longitudinal development of VEMP responses might aid the differential diagnosis of the two disorders.
